# The Expression of the Endogenous mTORC1 Inhibitor Sestrin 2 Is Induced by UVB and Balanced with the Expression Level of Sestrin 1

**DOI:** 10.1371/journal.pone.0166832

**Published:** 2016-11-18

**Authors:** Veronika Mlitz, Gaelle Gendronneau, Irina Berlin, Maria Buchberger, Leopold Eckhart, Erwin Tschachler

**Affiliations:** 1 Research Division of Biology and Pathobiology of the Skin, Department of Dermatology, Medical University of Vienna, Vienna, Austria; 2 Department of Biology and Women Beauty, Chanel R&T, Pantin, France; University of Alabama at Birmingham, UNITED STATES

## Abstract

Sestrin 2 (SESN2) is an evolutionarily conserved regulator of mechanistic target of rapamycin complex 1 (mTORC1) which controls central cellular processes such as protein translation and autophagy. Previous studies have suggested that SESN2 itself is subjected to regulation at multiple levels. Here, we investigated the expression of SESN2 in the skin and in isolated skin cells. SESN2 was detected by immunofluorescence analysis in fibroblasts and keratinocytes of human skin. Differentiation of epidermal keratinocytes was not associated with altered SESN2 expression and siRNA-mediated knockdown of SESN2 did not impair stratum corneum formation *in vitro*. However, SESN2 was increased in both cell types when the expression of its paralog SESN1 was blocked by siRNA-mediated knock down, indicating a compensatory mechanism for the control of expression. Irradiation with UVB but not with UVA significantly increased SESN2 expression in both keratinocytes and fibroblasts. Upregulation of SESN2 expression could be completely blocked by suppression of p53. These results suggest that SESN2 is dispensable for normal epidermal keratinization but involved in the UVB stress response of skin cells.

## Introduction

The homeostasis of cells is partly controlled by regulatory protein complexes, among which mechanistic target of rapamycin complexes (mTORC) have central roles. mTORCs respond to changes in intracellular concentrations of amino acids and stimulation by growth factors. They activate protein translation, suppress autophagy and indirectly regulate cellular homeostasis in organismal development and aging [1]. mTOR is inhibited by rapamycin, a macrolide drug also known as sirolimus [2], but also by endogenous inhibitors. Most notably, sestrins (SESNs) have been identified as evolutionarily conserved suppressors of mTOR signaling [3, 4].

Sestrins are implicated in sensing the concentration of the amino acid leucine, interacting with the TORC1-regulating GATOR2 protein complex, and the control of TORC1 trafficking to lysosomes, thereby ultimately altering the activity status of mTOR complexes [3–11]. The genes encoding sestrins (*SESNs*) are induced by various types of stress including oxidative stress [11–13], mitochondrial dysfunction [14, 15], endoplasmic reticulum stress [16] and DNA damage [5, 11]. While invertebrate model species such as *C*. *elegans* and *D*. *melanogaster* have only one sestrin gene [17, 18], mammals have three (*SESN1*, *SESN2* and *SESN3*). The physiological functions of sestrins have not been fully elucidated yet. Sestrin knock-down studies have shown that sestrin prolongs the lifespan and reduces age-related pathologies in *C*. *elegans* and *D*. *melanogaster*, respectively [17, 18]. Critical roles of sestrins in the regulation of the mammalian metabolism have been revealed by studies on *Sesn2* knockout as well as *Sesn2/Sesn3* double knockout mice [19]. Deletion of all three sestrins is incompatible with the survival of mice [3].

The skin is exposed to various types of environmental stress; and one of the most important is UV radiation. Depending on its type, UV-radiation has different effects in the skin. Whereas the less energetic UVA (320–400 nm) is considered to provoke mainly oxidative stress, contributing to skin aging and photocarcinogenesis [20], UVB (290–320 nm) causes DNA-damage leading to sunburn and skin cancer initiation as well as photoimmunosuppression favoring tumor spread [21]. Recently, it was shown that several pathways of the cutaneous neuroendocrine system are activated by UV radiation to counteract its deleterious effects. The former acts not only locally by regulating e.g. the skin barrier function and increasing the pigment production, but contributes also to systemic effects such as the immunosuppressive activity and the attenuation of autoimmune processes [22–27].

The skin undergoes constant self-renewing to maintain its essential function as a barrier to the environment. mTOR signaling has been recognized as an important point of control for several types of skin cells [28] and various approaches for therapeutic targeting of mTOR are already in clinical use or in different stages of development [29–31]. However, the roles of endogenous mTOR regulators of the SESN family in the skin have been only incompletely studied so far. SESN2 was recently implicated in the UV responses of skin cells and in the suppression of tumorigenesis [32], yet the control of its expression has remained unknown.

Here, we investigated the SESN2 expression in human skin fibroblasts and keratinocytes. Our data suggest that SESN2 is expressed in both keratinocytes and fibroblasts, its expression increases upon suppression of its paralog SESN1 and it is upregulated during the UVB stress response of skin cells.

## Results

### Sestrin 2 is expressed in fibroblasts and keratinocytes

**Fig 1:**
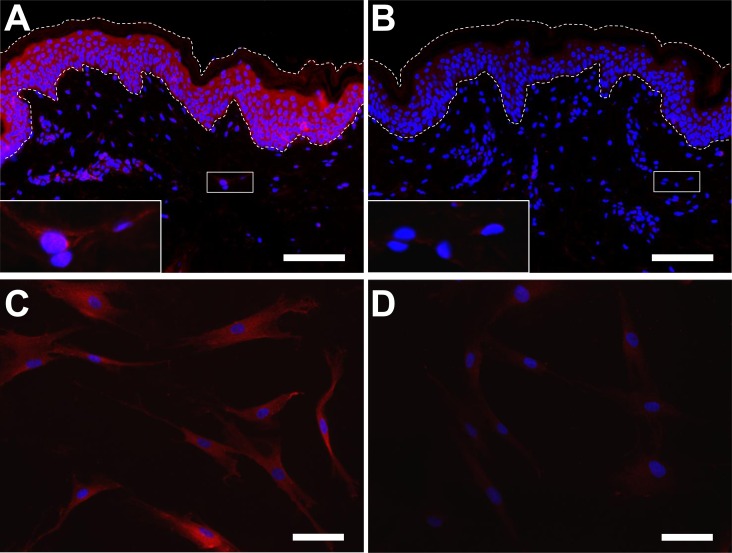
Expression of SESN2 in the skin. Human skin sections **(A, B)** or *in vitro* cultured primary human dermal fibroblasts **(C, D)** were immunolabeled with anti-SESN2 (red) either without **(A, C)** or with **(B, D)** preabsorption of the antibody with the antigen. Inhibition of labeling by antigen preabsorption is a negative control reaction to confirm the specificity of the antibody. Insets in A and B show higher magnification of dermal cells from the boxed areas of the sections. The dermo-epidermal junction and the surface of the epidermis are indicated by dotted lines. Bars: A and B, 100 μm; C and D, 50 μm.

The expression of SESN2 was determined by immunofluorescence analysis with an established antibody against amino acid residues 132–480 of human SESN2 [19, 33]. Immunofluorescence analysis showed a uniform labelling of dermal and epidermal cells that could be blocked by pre-adsorption of the antibody with the recombinant antigen, suggesting specific binding (Fig 1A and 1B). As the immunolabeling of dermal cells was weak on skin sections, we also performed immunolabeling for SESN2 in cultured primary human dermal fibroblasts. All fibroblasts of the cultures were immunopositive for SESN2 (Fig 1C and 1D).

**Fig 2:**
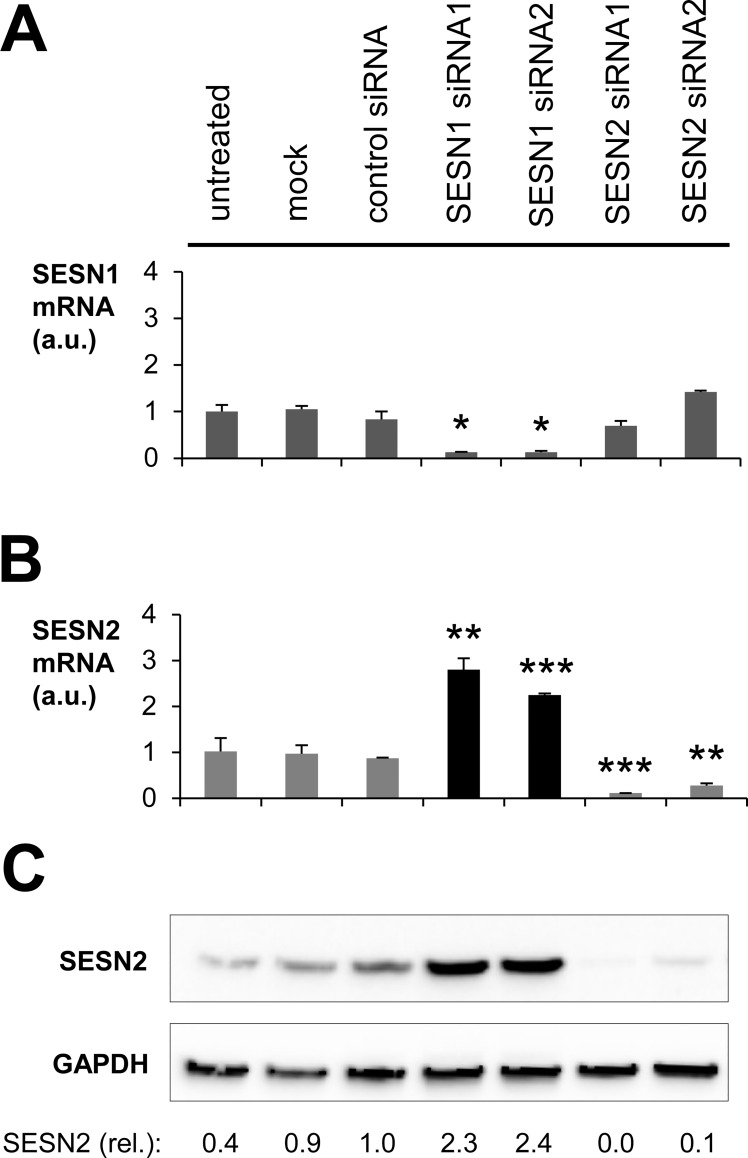
Compensatory upregulation of SESN2 upon knockdown of SESN1 in fibroblasts. Human primary fibroblasts were cultured in triplicates and transfected with siRNAs directed against SESN1 or SESN2. 48 h after the transfection, cells were harvested, RNA was extracted, transcribed into cDNA, and subjected to quantitative PCRs for SESN1 **(A)** as well as SESN2 **(B)**. Arbitrary units (a.u.) were calculated by normalizing the mRNA levels of SESN1 **(A)** or SESN2 **(B)** to B2M levels. Values relative to the expression levels in untreated cells are shown. Error bars indicate standard deviations. Student’s t-test was performed for comparisons between each treatment and the control siRNA treatment. *p < 0.05, **p < 0.01, ***p < 0.001. SESN2 protein expression was determined by Western blot analysis **(C)**. The band intensities, normalized to the intensities of the GAPDH bands on the same blot and relative to the control siRNA-treated samples, are indicated below the blot. The results are representative for three independent experiments using cells from different donors.

Western blot analysis of isolated epidermal keratinocytes and dermal fibroblasts confirmed expression of SESN2 in both types of skin cells. The specificity of the antibody was confirmed by the detection of a band at the expected size of SESN2 and by siRNA-mediated knockdown of SESN2 which abolished this band (Figs 2C and S1C).

To test whether SESN2 is required for survival and differentiation of keratinocytes *in vitro*, we knocked down SESN2 in proliferating cells, maintained them in culture for 1 more week, and induced stratification in an *in vitro* skin equivalent system [34]. SESN2-depleted keratinocytes differentiated normally and formed a normal stratum corneum *in vitro* (S2 Fig). These results suggested that SESN2 is not required for the terminal differentiation of epidermal keratinocytes.

### siRNA-mediated knock down of sestrin 1 leads to upregulation of sestrin 2 expression

To investigate the possible interdependence of expression levels of individual sestrins, these genes were knocked down by specific siRNAs in fibroblasts (Fig 2) and keratinocytes (S1 Fig). Only SESN1 and SESN2 were knocked down in fibroblasts because SESN3 was not detectable in appreciable amounts even in the basic state in these cells. Suppression of SESN1 expression triggered an upregulation of SESN2 mRNA (Fig 2A and 2B) and SESN2 protein (Fig 2C) in fibroblasts. In keratinocytes, SESN2 mRNA levels were not significantly altered upon knockdown of SESN1 and SESN3 (S1A and S1B Fig), but the amount of SESN2 protein increased strongly upon downregulation of SESN1 or SESN3 (S1C Fig), indicating a post-transcriptional mechanism of SESN2 induction under these conditions. Combinatorial knockdowns and the complete knockdown of all 3 sestrins could not be faithfully confirmed in our experimental system. Thus, it remains possible that the downregulation of SESN2 is compensated by one or both of the other sestrins in keratinocytes. Evidently, compensatory regulation of sestrin expression levels is active in both fibroblasts and keratinocytes.

### UVB irradiation causes increased expression of sestrin 2 in fibroblasts and keratinocytes

**Fig 3:**
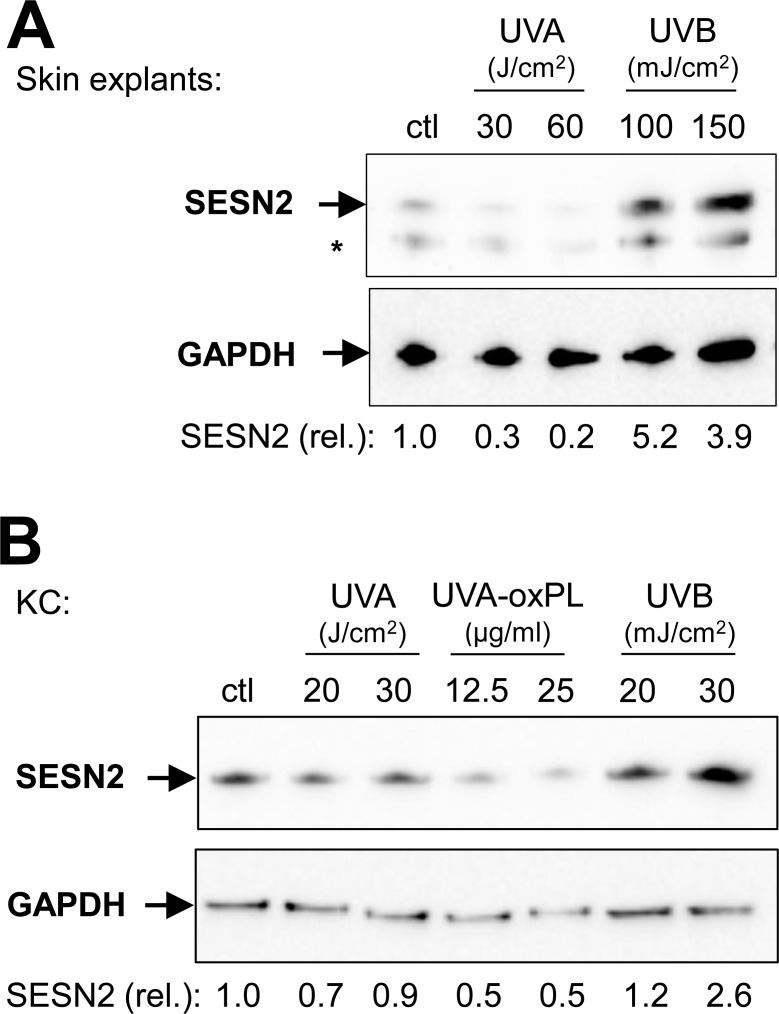
UVB induces SESN2 protein in isolated keratinocytes and skin explants. Human skin explants **(A)** and cultured keratinocytes (KC) **(B)** were irradiated with the indicated doses of UVA (J/cm^2^) or UVB (mJ/cm^2^) or treated with UVA-induced oxidized phospholipids (UVA-oxPL, μg/ml). 24 h after treatment, cells were lyzed and Western blots for SESN2 and GAPDH were performed. Bands at the predicted sizes of SESN2 and GAPDH are indicated. An asterisk marks an unidentified band immunoreactive with anti-SESN2. The expression of SESN2 relative to GAPDH (lower panels) was normalized to the non-irradiated control. One of three independent experiments with similar results is shown.

**Fig 4:**
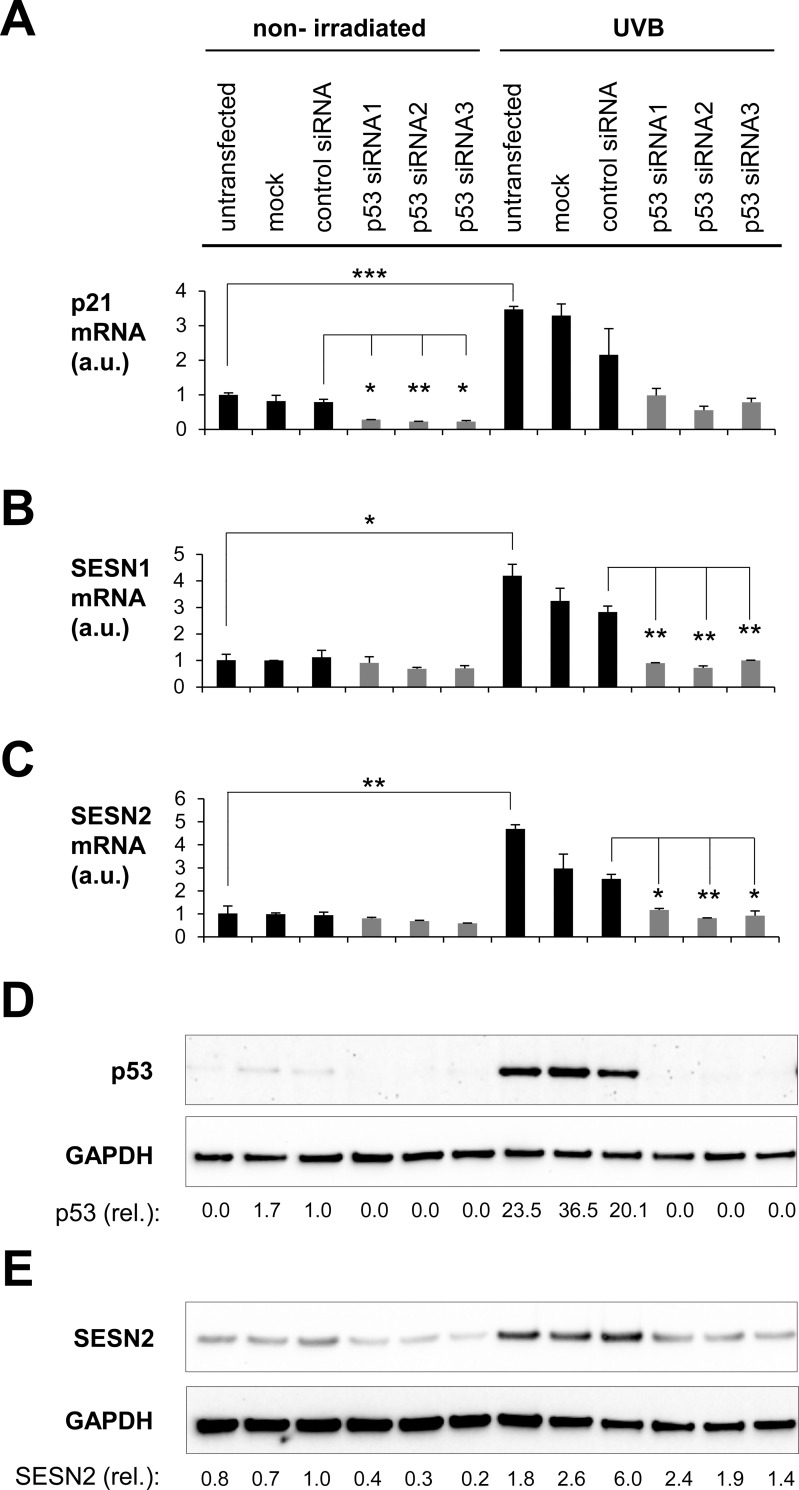
UVB induces upregulation of SESN1 and SESN2 via p53. Fibroblasts (FB) were treated with p53 siRNA, 48 h after transfection fibroblasts were irradiated with UVB 20 mJ/cm^2^. RNA was extracted 12 h later to monitor the expression levels of the p53 target gene p21 **(A)**, SESN1 **(B)** and SESN2 **(C)** at the mRNA level by RT-qPCR, relative to untreated cells, normalized to B2M. Error bars indicate standard deviations. For comparisons of untransfected non-irradiated versus untransfected UVB-irradiated cells and siRNA-treated versus control siRNA-treated cells (each within the non-irradiated and the irradiated groups), Student’s t-test was calculated, with p<0.05 being considered significant. *p < 0.05, **p < 0.01, ***p < 0.001. Protein lysates were prepared 8 h **(D)** and 24 h **(E)** post-irradiation to analyze the expression of p53 **(D)** and SESN2 **(E)** by Western blot. In each case, the membrane was re-probed with anti-GAPDH. The expression levels of SESN2 (relative to the non-irradiated control siRNA transfected cells, normalized to GAPDH) are indicated. One representative experiment of three independent experiments with cells of different donors is shown.

To investigate the effect of UV-induced stress on SESN2 expression, skin explants (Fig 3), keratinocytes (Figs 3 and S3) and fibroblasts (Figs 4 and S3) were irradiated with UVA or UVB. While UVA irradiation (20 J/cm^2^) had no discernible effect on SESN1-3 mRNA expression, UVB irradiation (20 mJ/cm^2^) led to an increase of SESN1 and SESN2 (S3 Fig) but not SESN3 mRNA in fibroblasts and keratinocytes. At the protein level, SESN2 protein was upregulated by UVB irradiation (100 and 150 mJ/cm^2^) of skin explants (Fig 3A). UVB (20 mJ/cm^2^) also induced an increase of SESN2 protein abundance in cultured keratinocytes (Fig 3B) and fibroblasts (Fig 4E).

Previous studies of cells from various tissues suggested that the induction of SESN1 and SESN2 in response to genotoxic stress depends on the transcription factor p53 [5, 35]. To investigate whether p53 is also involved in the UVB-induced upregulation of SESN1 and SESN2 in skin cells, p53 was knocked down in these cells *via* siRNA before UVB irradiation. In keratinocytes, expression of SESN1 and SESN2 was increased by UVB doses higher than 20 mJ/cm^2^. Treatment of keratinocytes with p53 siRNAs before the exposure to these doses was lethal to the cells. Therefore, further experiments were performed on fibroblasts. p53 siRNAs reduced the expression levels of the p53 target gene p21 in non-irradiated and UVB treated fibroblasts as compared to control-siRNA transfected cells (Fig 4A). Moreover, the p53 protein was induced 8 h post irradiation in control siRNA-transfected fibroblasts but not in cells treated with p53 siRNA and UVB (Fig 4D). These results confirmed an efficient knockdown of p53 by siRNAs. The p53 knock-down inhibited the upregulation of SESN1 and SESN2 at mRNA (Fig 4B and 4C) and protein levels (Fig 4E) in UVB-irradiated fibroblasts. Therefore, the induction of SESN1 and SESN2 in UVB-irradiated fibroblasts is mediated by p53.

## Discussion

The results of this study demonstrate that SESN2 is constitutively expressed in human fibroblasts and keratinocytes and suggest that its expression is further upregulated by UVB irradiation. As SESN2 regulates mTORC1 signalling, these data indicate a link between UV stress of the skin and alterations of mTORC1-dependent processes in the homeostasis of skin cells.

Gene-specific siRNA treatments in cultured fibroblasts and keratinocytes showed that, at least under specific experimental settings, SESN2 expression increases when expression of SESN1 is decreased and *vice versa*. This finding is compatible with the hypothesis that SESN1 and SESN2 are functional equivalents [7] and that cells try to compensate the lack of one sestrin by the upregulation of the other. The molecular signalling underlying this regulation remains to be determined. Recently, SESN1 and SESN2 but not SESN3 were identified as sensors for the intracellular concentration of leucine [7] which is required to stimulate mTORC1 activity [36]. Thus, signals from both SESN1 and SESN2 are likely to contribute to the regulation of mTORC1 activity *via* leucine. However, there may be as-yet unknown sensory and signalling functions that are specific to each SESN isoform.

To restore skin homeostasis in response to UVB induced harm, several signalling cascades such as the cutaneous neuroendocrine hypothalamic-pituitary-adrenal axis and pathways resulting in the stabilisation of p53 are activated [27, 37–40]. The tumor suppressor p53 has an important cellular gatekeeper function maintaining genomic stability in response to cellular stress, such as UVB induced DNA-damage, by inducing DNA-repair or apoptosis [38]. Our UV irradiation experiments suggest that sestrins also participate in the molecular UVB response of skin cells. Interestingly, UVA did not regulate the expression of any sestrin in keratinocytes and fibroblasts although oxidation products generated by UVA are relevant for processes such as mTOR signalling and autophagy which are under direct or indirect control by sestrins. By contrast, UVB induced a pronounced upregulation of SESN1 and SESN2 in fibroblasts, keratinocytes and skin explants (Figs 3 and 4E). The finding that, different from the effects of individual gene knockdown (Fig 2), SESN1 and SESN2 were upregulated in parallel by UVB (Fig 4) might indicate that, under conditions of severe UVB stress, both SESNs are needed to assure an appropriate stress-response. The UVB-inducible expression of SESN2 in this study is in line with previous reports on SESN2 expression in keratinocyte cell lines and primary skin cells [32, 41]. Recently, SESN2 was reported to be upregulated by UVB, but also by UVA, in melanocytes [42]. Our knockdown experiments in fibroblasts demonstrated that UVB-induced upregulation of SESN2 depends on p53, which is compatible with the presence of a functional p53 binding sites in the promoter and the first intron of SESN2 [43, 44]. Of note, SESN2 has been reported to be under additional regulation by the stress response regulators Nrf2 and JNK in various cell types [12, 45]. Therefore, also in skin cells SESN2 is likely to be controlled not only by p53 but also by other signalling pathways that remain to be investigated.

Several aspects of sestrin regulation in the skin remain to be addressed in future studies. While the expression of sestrins was studied here mainly in cultures of isolated skin cells, interactions between different cell types may affect sestrin expression and this may be relevant for the roles of sestrins *in vivo*. Furthermore, the cutaneous expression of SESN1 and SESN3 should be investigated not only at the mRNA but also the protein level which will require the generation and validation of suitable antibodies. It will be interesting to determine whether stress-dependent alterations of the expression levels of SESN2 affect recently reported SESN2-dependent responses to changes in the intracellular leucine concentration [7, 9]. The results of the present study provide the basis for evaluating the link between the regulated expression and functions of SESN2 in the skin.

## Material and Methods

### Ethics statement

The local ethics committee at the Medical University of Vienna (EK2011/1149) approved the use of skin samples obtained from plastic surgery for all experiments performed in this study. All donors provided written informed consent.

### Cell culture

Fibroblasts and keratinocytes were isolated from fresh skin samples of donors aged between 22 and 65 years. For separation of epidermis and dermis, the tissue samples were incubated with 2.4 U/ml dispase (Roche Applied Science, Basel, Switzerland) at 4°C overnight. The epidermis was further incubated for 8 min at 37°C with trypsin (Lonza, Basel, Switzerland). After addition of DNase1 the cell suspension was filtered. Isolated keratinocytes were grown in KGM2 medium (Lonza). The dermis was treated for 1 h at 37°C with collagenase (Thermo Fisher Scientific, Waltham, MA) and filtered several times. Cells isolated from the dermis were grown in DMEM supplemented with penicillin/streptomcycin (Thermo Fisher Scientific) and fetal bovine serum (Biochrom AG, Berlin, Germany).

### siRNA mediated knockdown of gene expression

Knock-down experiments were performed with stealth™ siRNAs (Thermo Fisher Scientific) according to previously published protocols [46, 47]. Transfected keratinocytes were either used directly for RNA and protein extraction or for organotypic skin cultures. A detailed protocol of the latter can be found in previously published studies [34, 47]. Briefly, 1.5 x 10^6^ keratinocytes were seeded on collagen type I (Biochrom, Berlin, Germany) containing 1 x 10^5^ fibroblasts in 3 μm pore sized cell-culture inserts (Corning Life Sciences, Tewksbury, MA). After 7 days at air liquid interphase, supplemented with serum-free keratinocyte defined medium, consisting of KGM2 (Lonza) without bovine pituitary extract and epinephrine, but with 1.5 mM calcium (Sigma), 50 μg/ml ascorbic acid (Sigma) and 0.1% bovine serum albumin (Sigma), organotypic skin cultures were harvested. SESN2 knockdown experiments in organotypic skin cultures were performed with two different siRNAs in duplicates.

### UV irradiation

For irradiation experiments, cells were seeded in 6-well and 12-well plates. Prior to irradiation, cells were washed with phosphate-buffered saline (PBS), pH 7.4 (Thermo Fisher Scientific). Cells were irradiated under a thin layer of PBS at 25°C. Fresh skin explants were placed in 6-well plates containing PBS and irradiated directly. As a light source for UVA, a Sellamed 3000-type solar simulator (Sellas Medizinische Geräte, Ennepetal, Germany) filtered for the emission of UVA (340–440 nm) was used [48]. Its spectrum is described in a previous study [49]. UVB (280–320 nm) irradiation was performed with a Waldmann F15 T8 tube (Waldmann Medizintechnik, Villingen-Schwenningen, Germany), the spectrum is found in a previously published protocol [50]. The irradiance at a tube to target distance of 30 cm for the UVA and UVB source was 66 mW/cm^2^ or 1 mW/cm^2^, respectively. The times of irradiation were calculated using the formula: time (s) = dose (J/cm^2^)/intensity (W/cm^2^) [51]. Doses of irradiation were UVA: 0, 20, 30 and 60 J/cm2 (irradiation time: 0, 5, 7.5 and 15 min) and UVB: 0, 20, 30, 100 and 150 mJ/cm2 (irradiation time: 0, 20, 30, 100 and 150 sec). Immediately after irradiation, PBS was removed and cells were incubated with serum-free basic medium. In other experiments, instead of direct UVA irradiation of cells, UVA was administered to phospholipids to generate UVA-induced oxidized phospholipids (UVA-oxPL) which were subsequently applied onto cells according to published protocols [52].

### RNA preparation, cDNA synthesis, and quantitive PCR (qPCR)

RNA was purified from homogenized tissues (Precellys, VWR International, Radnor, PA) with TriFast (VWR International) according to the manufacturer´s instructions. Stress stimulated cells were lysed with 300 μL RLT buffer (Qiagen, Hilden, Germany) and RNA was extracted using the RNeasy kit (Qiagen) according to the manufacturer’s protocol. RNA was reverse-transcribed to cDNA with the Iscript^TM^ Kit (Biorad, Hercules, CA). The qPCR was performed using the LightCycler® technology (LC480) and the LightCycler 480 DNA SYBR Green I Master Kit (Roche Applied Science) according to the manufacturer’s protocol. The following primers were used: b2m-f (5´-gatgagtatgcctgccgtgtg-3´) and b2m-r (5´-caatccaaatgcggcatct-3´) for the housekeeping gene *Beta-2 microglobulin* (*B2M*); SESN1-f (5´-agcccatagaccttggctta-3´) and SESN1-r (5´-tccacactgtgattgccatt-3´) for *SESN1*; SESN2-f (5´-tgctgtgctttgtggaagac-3´) and SESN2-r (5´-gctgcctggaacttctcatc-3´) for *SESN2*.

### Statistical analysis

To access the statistical significances of the qPCRs, homoscedastic two-tailed, Student’s t-tests were performed between control siRNA transfected cells and other treated cells or between non-irradiated and UV-treated cells. p<0.05 was considered significant and significance was indicated as follows: *p < 0.05, **p < 0.01 and ***p < 0.001.

### Protein preparation and Western blot analysis

Proteins were extracted from cells by sonication and from tissues with the Precellys homogenizer (Peqlab) in a buffer containing 2% SDS. Protein concentrations were measured with the Micro BCA Protein Assay Kit (Thermo Fisher Scientific). 10–30 μg protein per lane were electrophoresed through an ExcelGel SDS gradient 8–18% polyacrylamide gene (GE Healthcare Life Sciences, Chicago) and blotted onto a nitrocellulose membrane. The membranes were incubated with the primary antibodies at 4°C over night. The following antibodies were used: mouse monoclonal anti-p53, clone DO-1 (1: 500, OP43, lot D32095, Calbiochem, Merck, Darmstadt, Germany), affinity-purified polyclonal rabbit anti-SESN2 (1:1000, 10795-1-AP, lot 00016483, Proteintech, Rosemont, IL) and mouse monoclonal anti-GAPDH (1:2000, 5G4 MAb 6C5, lot 15/04-G4-C5, HyTest Ltd, Turku, Finland). The specificity of the immunoreactions was confirmed by using antibodies that were preabsorbed with the antigen. The second step antibodies coupled with HRP were incubated for 1 h at room temperature. Finally, the target proteins were visualized using the ECL system (Thermo Fisher Scientific).

### Immunofluorescence and histological analysis

Sections of tissues, fixed with 7.5% formaldehyde and embedded in paraffin, were incubated with rabbit anti-SESN2 antibody (1:2000; Proteintech). Goat anti-rabbit immunoglobulin conjugated to Alexa-Fluor 546 (1:500, A11010, lot 1691774; Thermo Fisher Scientific) was used as secondary antibody. Goat serum (10%) was added to the secondary antibody to suppress unspecific binding. Labeling of nuclear DNA was performed with Hoechst 33258 (Thermo Fisher Scientific). To confirm the specificity of the immunolabeling, an antibody preabsorption experiment was performed. The primary antibody (anti-SESN2, Proteintech) was incubated at a concentration of 0.1 μg/ml with 0.2 μg of the immunogen (a human SESN2-glutathione S-transferase (GST) fusion protein encompassing amino acid 132–480 of human sestrin 2, NP_113647.1, Ag 1247, lot 30141134, Proteintech), against which the antibody was raised, in a total volume of 20 μl PBS in the presence of 2% bovine serum albumin (Sigma-Aldrich, St Louis, MO). After 2 hours at 4°C, the preincubation solution was diluted further so that the immunolabeling was done at a final antibody dilution of 1:2000. In the actual SESN2 immunolabeling experiments, the primary antibody was preincubated with PBS/bovine serum albumin without antigen. Hematoxylin and eosin staining was performed according to a standard protocol [53].

## Supporting Information

S1 Fig Knockdown of SESN1 increases SESN2 protein abundance in keratinocytes and knockdown of SESN2 confirms specificity of the SESN2 immunoblot. Human keratinocytes were transfected with siRNAs against SESN1, SESN2 and SESN3. 48 hours after transfection cells were harvested, and RNA was extracted, transcribed into cDNA and subjected to qPCRs for SESN1 **(A)** and SESN2 **(B)**. Protein lysates were subjected to SESN2 Western blot analysis **(C)**. The expression of SESN2 protein relative to control siRNA-treated cells was normalized to the amount of GAPDH. *p < 0.05, **p < 0.01, Student’s t-test, between treatments with specific siRNAs and control siRNA.
(PDF)

S2 Fig siRNA-mediated knockdown of SESN2 does not impair terminal differentiation keratinocyte. Organotypic skin equivalents were made with keratinocytes transfected with a control siRNA **(A)** or a siRNA directed against SESN2 **(B)**. Sections were stained with hematoxylin and eosin (H&E). Note the formation of an eosinophilic (red) stratum corneum (sc) in both cultures. Differences in the thickness of the epidermal component are within the normal range of variability. The SESN2 knockdown was performed with 2 different SESN2-specific siRNAs, each in duplicates, with similar results. epi, epidermal component; derm, dermal component (collagen matrix and fibroblasts). Bars, 100 μm.
(PDF)

S3 Fig UVB irradiation induces SESN1 and SESN2 mRNA in fibroblasts and keratinocytes. Human fibroblasts **(A, B)** and keratinocytes **(C, D)** were irradiated either with 20 J/cm^2^ UVA or with 20 mJ/cm^2^ UVB. Cells were harvested at the indicated timepoints. RNA was extracted, transcribed into cDNA and SESN1 and SESN2 qPCRs were performed. The bars indicate the mean values for cells isolated from three different donors. Error bars indicate standard deviations. *p < 0.05, **p < 0.01, Student’s t-test between control and irradiated cells.
(PDF)
