# Supplementary figures and images for: The Expression of the Endogenous mTORC1 Inhibitor Sestrin 2 Is Induced by UVB and Balanced with the Expression Level of Sestrin 1
Source: PLoS One. 2016 Nov 18;11(11):e0166832. doi: 10.1371/journal.pone.0166832 (PMC5115827; doi:10.1371/journal.pone.0166832)

**A**

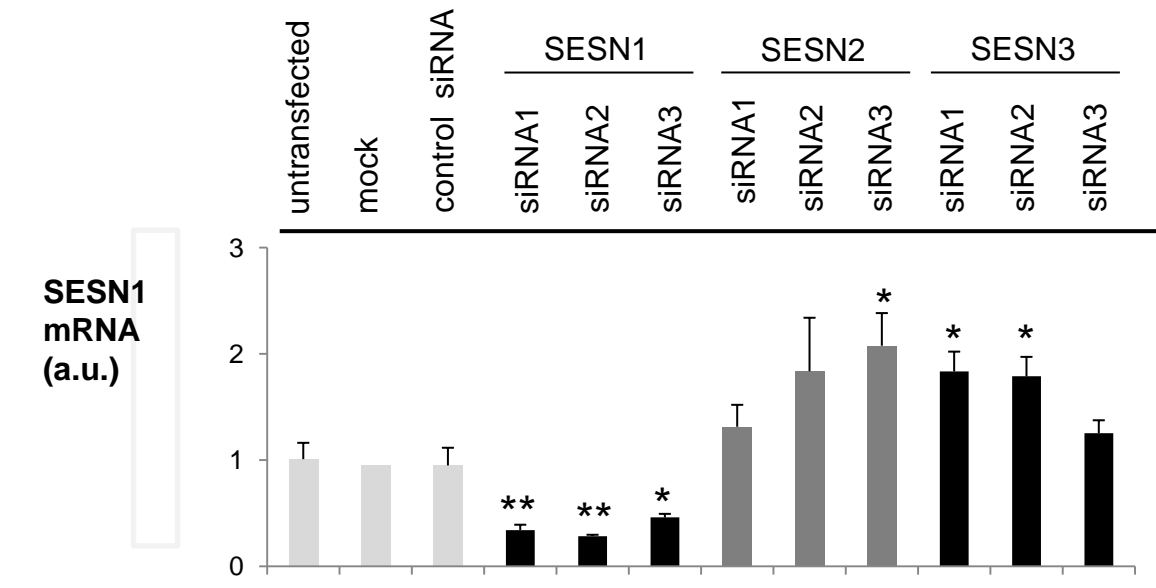

**B**

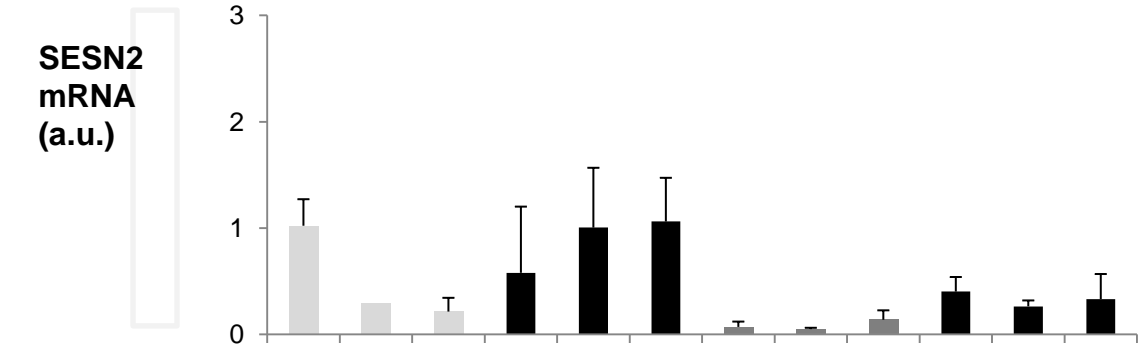

**C**

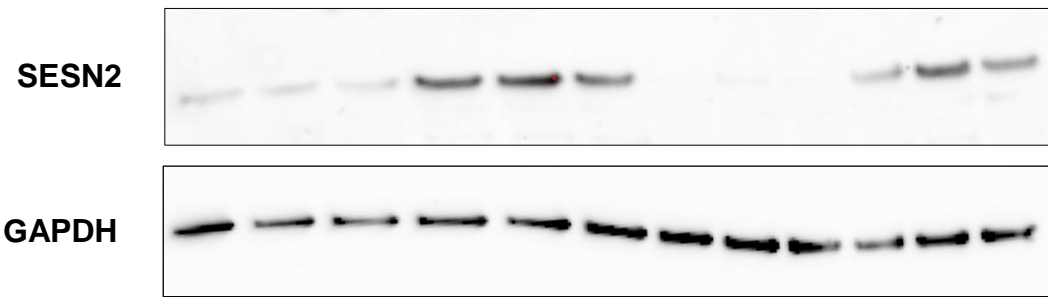

Supplement: S1 Fig — Human keratinocytes were transfected with siRNAs against SESN1, SESN2 and SESN3. 48 hours after transfection cells were harvested, and RNA was extracted, transcribed into cDNA and subjected to qPCRs for SESN1 (A) and SESN2 (B). Protein lysates were subjected to SESN2 Western blot analysis (C). The expression of SESN2 protein relative to control siRNA-treated cells was normalized to the amount of GAPDH. *p < 0.05, **p < 0.01, Student’s t-test, between treatments with specific siRNAs and control siRNA. (PDF) [file pone.0166832.s001.pdf]

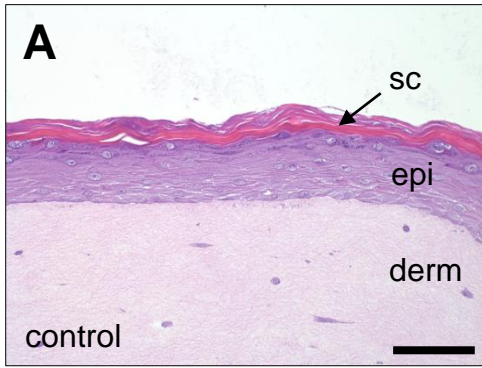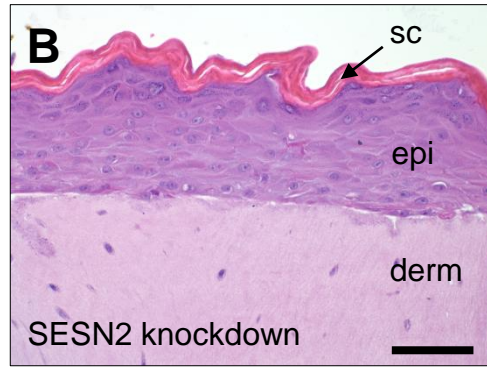

Supplement: S2 Fig — Organotypic skin equivalents were made with keratinocytes transfected with a control siRNA (A) or a siRNA directed against SESN2 (B). Sections were stained with hematoxylin and eosin (H&E). Note the formation of an eosinophilic (red) stratum corneum (sc) in both cultures. Differences in the thickness of the epidermal component are within the normal range of variability. The SESN2 knockdown was performed with 2 different SESN2-specific siRNAs, each in duplicates, with similar results. epi, epidermal component; derm, dermal component (collagen matrix and fibroblasts). Bars, 100 μm. (PDF) [file pone.0166832.s002.pdf]

**A**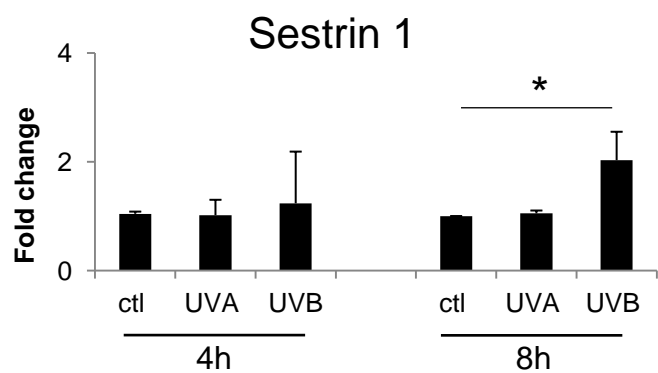**B**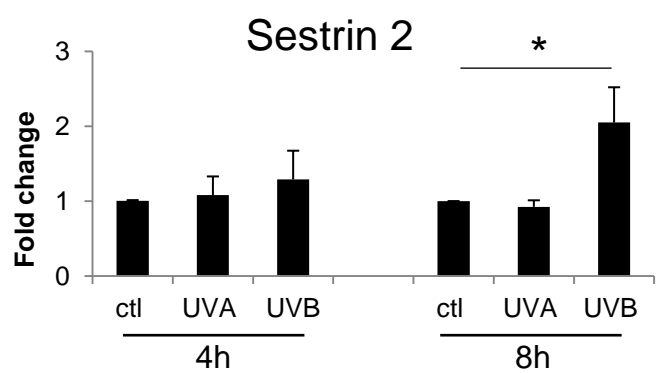**C**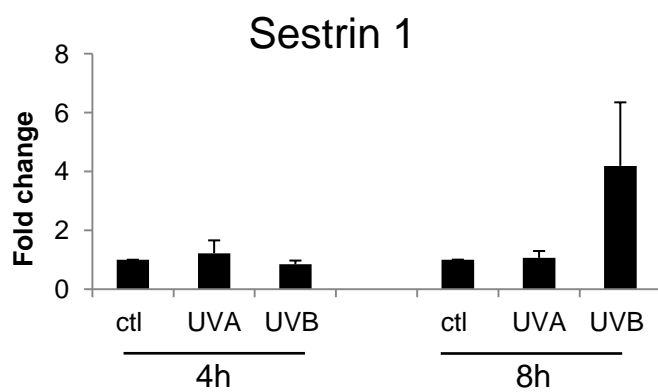**D**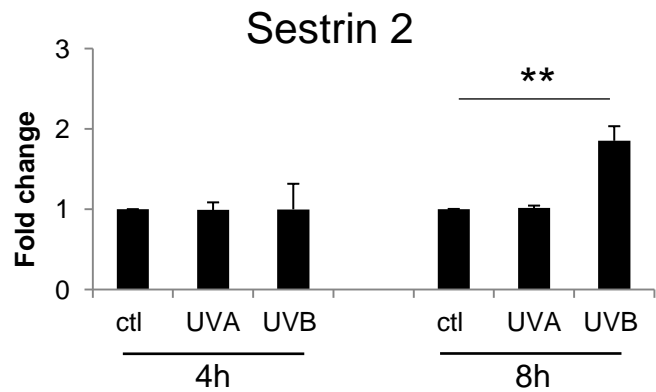

Supplement: S3 Fig — Human fibroblasts (A, B) and keratinocytes (C, D) were irradiated either with 20 J/cm2 UVA or with 20 mJ/cm2 UVB. Cells were harvested at the indicated timepoints. RNA was extracted, transcribed into cDNA and SESN1 and SESN2 qPCRs were performed. The bars indicate the mean values for cells isolated from three different donors. Error bars indicate standard deviations. *p < 0.05, **p < 0.01, Student’s t-test between control and irradiated cells. (PDF) [file pone.0166832.s003.pdf]
